# *Cupressus arizonica*: an emerging aeroallergen for East Mediterranean children

**DOI:** 10.55730/1300-0144.5692

**Published:** 2023-07-11

**Authors:** Hilal ÜNSAL, Ümit Murat ŞAHİNER, Özge SOYER, Bülent Enis ŞEKEREL

**Affiliations:** Department of Pediatric Allergy, Faculty of Medicine, Hacettepe University, Ankara, Turkiye

**Keywords:** Aeroallergen, children, *Cupressus arizonica*, Cup a 1, nasal allergen challenge, respiratory

## Abstract

**Background/aim:**

*Cupressus sempervirens* is a tree native to the Mediterranean region. We aimed to investigate the frequency of sensitization/allergy to *Cupressus arizonica* pollen, which is not native to Anatolia.

**Materials and methods:**

Patients aged 5–18 years who underwent respiratory allergy screening in Türkiye’s largest referral center over a 1-year period were reviewed retrospectively for a diagnostic study of *Cupressus* allergy.

**Results:**

Of 246 patients, 207 (67.6% male) with a median age of 11.7 (IQR 9.2–15) years were found to be aeroallergen-sensitive and *C. arizonica* (32%) was the second most common sensitivity after grass pollen (83.6%). In the *C. arizonica*-sensitive subgroup, only 3% (2/67) were monosensitive, and grass (77.6%), cat (38.8%), and weeds (38.8%) were the most common co-sensitivities. Cup a 1 specific IgE (sIgE) was measured in 26 patients with *C. arizonica* sensitivity and all were found to be positive. A nasal allergen challenge (NAC) was performed for 44 of 67 patients with *C. arizonica* sensitivity, and 13 of 44 patients had a positive outcome (NAC+) at the highest two extract concentrations. The *Cupressus* wheal sizes and Cup a 1 sIgE levels of the NAC+ subgroup were higher than those of the NAC– subgroup but reached significance only for wheal size [6 (5–7.5) vs. 4.5 (4–6), p=0.004]. The NAC+ subgroup reported more frequent nasal discharge, congestion, and eye symptoms than the NAC– subgroup during the relevant pollen season.

**Conclusion:**

*C. arizonica* sensitivity has increased in the East Mediterranean region, similarly to North Mediterranean data, and this is associated with the presence of allergy both clinically and in laboratory findings. *C. arizonica* should be included in the aeroallergen screening panels of children from the East Mediterranean.

## 1. Introduction

Tree pollen allergy is not a leading problem in Türkiye, except for olives in the southern and western coastal regions of the country. *Cupressus sempervirens* is a tree native to the Mediterranean region and has an olive-like geographic distribution. However, available data suggest that it is not a leading respiratory allergen [1,2]. *Cupressus arizonica* is not a native species in Anatolia and its use in landscaping is increasing due to its bluish color, low cost, and resistance to cold, heat, fire, and drought [3,4]. This increased cultivation may have resulted in an increased prevalence of sensitization or allergy.

Accurate allergy diagnosis is based on a detailed history, demonstration of sensitivity in allergy tests [skin prick test (SPT) and extract- and/or component-specific IgE (sIgE)], and, if necessary, demonstration of clinical reactivity in challenge tests. Extract-based measurements of SPTs and sIgE may result in different results depending on the characteristics of the allergen extract source and the method used. Cup a 1 is the main allergen identified from *C. arizonica* [5]. Measurements of component-specific sIgE are used in allergy practice, especially in the analysis of multisensitivity related to panallergen or cross-reactive carbohydrate sensitivity [6].

The nasal allergen challenge (NAC) is an important tool for diagnosing allergic rhinitis [7]. It can be used in a wide range of clinical practices, including distinguishing between sensitization alone and allergy, identifying appropriate patients for allergen immunotherapy, and identifying patients with local IgE in the absence of systemic sensitization [8]. A wide variety of techniques and interpretation tools have been proposed for the NAC, and several recommendations are available for its execution [9]. The NAC provides reliable results, but it is also time-consuming and costly [9]. With these properties, the NAC is not routinely used for diagnostic purposes, but it is valuable for assessing the presence of clinical reactivity when SPTs and sIgE measurements are not helpful [1].

Considering that the frequency of *Cupressus* allergy may change over time and that the results of detailed allergy tests may differ, we aimed to share our experiences of *Cupressus* allergy in the largest allergy reference center in Türkiye.

## 2. Materials and methods

### 2.1. Study design and subjects

This retrospective study was conducted in the Pediatric Allergy Division of Hacettepe University İhsan Doğramacı Children’s Hospital. Patients aged 5–18 years who were tested for respiratory allergies during a 1-year period (June 1, 2020 to July 30, 2021) were reviewed. Diagnoses of atopic dermatitis (AD), allergic rhinitis (AR), and asthma were made according to international guidelines [10–12]. Information on whether the patients had symptoms of AR during the relevant pollen season was obtained from patient files containing a questionnaire prepared by adapting the International Study of Asthma and Allergies in Childhood (ISAAC) Phase II questionnaire [13,14]. In addition to SPT results, the patients’ demographic data and all test results for *Cupressus* allergy, such as component sIgE measurements and NAC results, were also collected.

### 2.2. Skin prick tests

In the routine practice of the clinic, for SPTs, allergen extracts (ALK Pharmaceuticals, Mississauga, Canada) were applied to the volar surface of the forearm or back along with negative and positive controls as detailed previously [15]. Patients were tested with a panel of common aeroallergens [*Dermatophagoides pteronyssinus*, *Dermatophagoides farinae*, cat, dog, weed pollen mix (*Artemisia vulgaris*, *Chenopodium album, Parietaria judaica*, *Plantago lanceolata*, *Salsola kali*), *Alternaria alternata*, *Cladosporium herbarum*, tree pollen mix (*Betula alba*, *Corylus avellana*, *Olea europaea*, *Platanus*, *Populus*, *Salix*, *Quercus*, *Ulmus*), grass pollen mix (*Dactylis glomerata*, *Festuca rubra*, *Lolium perenne*, *Phleum pratense*, *Poa pratensis*, *Avena sativa*), *Cynodon dactylon*, *Blattella germanica*, and *Cupressus arizonica*] [16,17].

### 2.3. Measurements of component sIgE

For the relevant *C. arizonica* allergen molecule, Cup a 1 sIgE measurements were evaluated as either singleplex (Immuno-CAP, Thermo Fisher Scientific, Uppsala, Sweden) or multiplex ALEX^2^ (MacroArray Diagnostics, Vienna, Austria) measurements. The component sIgE for Cup a 1 was evaluated at the discretion of the consultant to confirm either a positive response to the SPT or the differential diagnosis of multiple sensitizations. In addition to Cup a 1 sIgE, the ALEX^2^ assays provided extract-specific IgE for *Cupressus sempervirens* and component sIgEs for *Cryptomeria japonica* (rCry j 1) [18].

### 2.4. Nasal allergen challenge with *C. arizonica*

The NAC is not routinely performed in the clinic. It was performed for a subgroup of patients at the discretion of the consultant to confirm whether *C. arizonica* allergy was a multiple-pollen sensitization and/or a low level of *C. arizonica/*Cup a 1 sensitivity. These NACs were conducted after the parents and children provided consent. For the allergen challenge, a *C. arizonica* allergen extract (Arizonica Cypress allergen 1:20 W/V, ALK Pharmaceuticals, Canada) was used. The instilled concentrations were obtained from raw extracts of *C. arizonica* (0.1 μg/mL, 1 μg/mL, 10 μg/mL, 100 μg/mL, and 1000 μg/mL). Spray bottles with a 50 μL/puff nozzle were used for allergen applications. The NAC started with physiological saline and increasing concentrations of the allergen were given every 10 min. The allergen was applied by administering 2 puffs of 0.05 mL per puff per nostril, one in the inferior meatus and one in the direction of the middle turbinate. The precise instructions were to take a deep breath before, hold the breath during, and exhale strongly after the application of the allergen [7].

Immediately before each subsequent dose, the participants were asked to grade their symptoms according to a verified scoring system including a total nasal symptom score and eye symptoms [7,19]. A positive result was noted for patients who reported symptom scores of >3. During the NAC, the exclusion criteria and precautionary measures recommended by the relevant guidelines were followed [7,9,19].

### 2.5. Ethics

The local ethics committee of the Hacettepe University Faculty of Medicine approved the study (Number: GO-21/762, Date: June 29, 2021).

### 2.6. Statistical analysis

Descriptive analysis was used to characterize the patients. The Pearson chi-square (χ^2^) test or Fisher exact test was used for between-group comparisons. Values were shown as medians and interquartile ranges for nonnormally distributed data. The Mann–Whitney U test or Kruskal–Wallis test was used to compare values. All statistical tests were two-sided and the level of statistical significance was set at p<0.05.

## 3. Results

### 3.1. Study population

During the study period, a total of 246 patients aged 5–18 years with a prediagnosis of respiratory allergy were evaluated for aeroallergen sensitization, and 207 were found to be sensitized, with a median age of 11.7 (IQR 9.2–15) years (67.6% male).

Of the 207 patients, 83.6% (n = 173) were sensitized to grass pollen (grass mix and/or *Cynodon* dactylon), 25.1% (n = 52) to weed pollen, 25.1% (n = 52) to cat, 17.4% (n = 36) to house dust mite, 16.9% (n = 35) to *Alternaria*, 11.6% (n = 24) to dog, and 32.3% (n = 67) to *C. arizonica*. Patients with one sensitization, two sensitizations, and three sensitizations respectively accounted for 39.1%, 22.2%, and 20.7% of the total.

Among the *C. arizonica*-sensitized patients, the median SPT score was 5 (IQR: 4–6) mm and male patients (n = 53, 79.1%) were predominant. In the *C. arizonica-*sensitive group, 29.8% had atopic dermatitis, 35.8% had asthma, and 89.5% had AR.

Only 3% (2/67) of the patients were monosensitive to *C. arizonica*, and grass pollen (77.6%), cat (38.8%), and weed pollen (38.8%) were the most common co-sensitivities, respectively (Figure). Cup a 1 sIgE was measured for 26 patients with sensitivity to *C. arizonica* by SPT and all were found to be sensitive to Cup a 1, either through singleplex (n = 10) or multiplex (n = 16) arrays (Table 1). For all of the 16 patients who underwent multiplex tests, Cup a 1 sensitivity was also associated with the *Cryptomeria japonica* component rCry j 1 [2.9 kUA/L (IQR: 0.7–6.4)] and the *Cupressus sempervirens* allergen extract [0.54 kUA/L (IQR: 0.2–1.2)] sensitivity.

### 3.2. Nasal allergen challenge with *Cupressus arizonica*

NACs were performed for 65.6% (44/67) of the patients with *C. arizonica* sensitivity. For 13 of 44 (29.5%) patients, the NAC yielded positive results (NAC+), with 23.1% (n = 3) and 76.9% (n = 10) being positive at the steps of 100 μg/mL and 1000 μg/mL, respectively. Sneeze occurred in 76.9% of these cases, nasal congestion in 69.2%, itchy nose in 30.8%, and ocular symptoms in 30.8%; no patients had lower respiratory symptoms.

Among the patients who underwent NAC, Cup a 1 levels were evaluated for a total of 19 patients, 10 by singleplex and 9 by multiplex arrays. Considering the Cup a 1 values, higher values were obtained for patients with positive NAC outcomes; however, these values did not reach statistical significance, possibly due to the low number of participants in each subgroup.

Of the patients who underwent NACs, 90.9% (40/44) reported symptoms of current rhinitis during the pollen season of *Cupressus*. Sneezing (100% vs. 71%), eye findings (84.6% vs. 49.5%), and mouth breathing (38.5% vs. 12.9%) were more frequent in the NAC+ subgroup compared to the NAC– subgroup (p < 0.05 for each) (Table 2). The *Cupressus* SPT levels of the NAC+ subgroup [6 (IQR: 5–7.5) mm] were statistically higher than those of the NAC– subgroup [4.5 (IQR: 4–6) mm] (p = 0.004) (Table 3).

## 4. Discussion

In this unique pediatric study focusing on *Cupressus* allergy in Türkiye, we have demonstrated sensitivity to *C. arizonica* through SPTs in 32.3% of patients with a history of respiratory allergy, and this sensitivity was mostly associated with multiple aeroallergen sensitivity. The Cup a 1 component was positive in all cases for which it could be performed, and NAC positivity was demonstrated in 29.5%. Positive NAC outcomes were associated with higher levels of wheal edema and Cup a 1 sIgE and with a relevant history of AR.

The extent of *Cupressus* allergy has not yet been fully defined, except in some Mediterranean countries (e.g., France, Italy, Spain, Greece, and Israel), Japan, and the southwestern part of the United States, due to the paucity of published data [1,20–22]. The available studies are mostly based on documentation of sensitivities, and their relationships with allergy based on the NAC and/or molecular allergology have not been studied often. The present study was a retrospective study, and the relative overuse of the NAC and component sIgE measures reflect the need for consultants to be persuaded about the presence of *Cupressus* allergy.

To date, few studies investigating sensitivity to *Cupressus* pollen have been conducted in Türkiye [1,16,23]. The first was conducted in 2008 among adults with seasonal AR in an area with *C. sempervirens* in the native vegetation, and the sensitivity rate was found to be 14.3%. However, serum sIgE against *Cupressus* was positive in only two-thirds of these cases and only one patient had a positive NAC result [1]. The authors concluded that most of the sensitization might be due to cross-reactivity between pollen species. Our group previously defined Türkiye’s optimal SPT panel in terms of the number and variety of allergen extracts required to detect allergen sensitivity in children and adolescents with respiratory symptoms. In that study, *Cupressus* extract was not recommended for inclusion on the panel [17]. A complementary study was conducted shortly thereafter, which showed that adding *Cupressus* pollen extract to the panel could increase the detection rate of sensitization, especially among patients with AR symptoms. In that newer study, the sensitization rate of *Cupressus* extract in children/adolescents sensitive to aeroallergens was found to be 7.5% [16]. However, the authors could not eliminate the possibility of potential cross-sensitivity, and further testing including component sIgE quantifications or challenge tests was recommended to define the clinical relevance. Although the present study was conducted in a different period, it is complementary to the previous two studies and shows an increase of more than fourfold in *Cupressus* sensitivity.

There are reports documenting the increasing use of *C. arizonica* in gardening and reforestation in Türkiye in recent years [3], as previously reported in other countries [24,25], with higher pollen loads due to climate change, as also reported previously [26]. An Italian multicenter study performed in 2014 found an unexpectedly widespread diffusion of cypress pollen allergy throughout the country [27]. In Milan, a total of 5626 patients were diagnosed as having pollen allergies in an outpatient clinic and, among the pollen allergy group, 1125 (20%) were found to be sensitized to cypress pollen [24]. Similarly, Caimmi et al. found that 20.7% of patients were sensitized to cypress pollen in the Montpellier area [28].

Measuring component sIgE has emerged as a promising tool for diagnosis, but there are several potential barriers such as the cost, necessity for multiple studies, social security coverage, and poorly defined panels of components. In our study, of 67 patients with *C. arizonica* sensitivity, 26 were investigated for Cup a 1 sIgE using singleplex or multiplex arrays and all were found to be positive. This implies that SPTs using *C. arizonica* pollen extract do not cause false positivity for *Cupressus* sensitivity and component-resolved diagnoses do not further improve SPT outcomes, given that only a subset of patients was investigated for Cup a 1 sensitivity. It should be emphasized here that the increased sensitivity of SPTs with commercial cypress pollen extracts was demonstrated after the previously used *C. sempervirens* was replaced with the much more allergenic *C. arizonica* as the source material [29].

Cross-reactivity is high in the cypress family. The group 1 major allergens belong to the pectate-lyase family and these members share 70% to 97% sequence homology within the different species of Cupressaceae. Those identified in all species of cypress include the number “1” in their IUIS reference names (e.g., Cry j 1, Cup s 1, Cup a 1, and Jun a 1) and are considered to be the major allergens of Cupressaceae, sensitizing almost 100% of patients who are allergic to cypress [18]. In our study, 16 patients underwent multiplex testing and, besides Cup a 1 sensitivity, all were also sensitive to rCry j 1 and extract-specific IgE for *C. sempervirens*, which confirms the existing high rate of homology.

An accurate diagnosis of respiratory allergy is essential to restore the patient’s quality of life. A thorough history, physical examination, and allergy testing (including SPTs and serum sIgE measurements) may lead to the diagnosis. NAC testing is believed to be a safe and simple technique, recommended for confirming the diagnosis, determining the phenotypes of the disease, and identifying allergens for which specific allergen immunotherapy should be administered [7]. However, the validity of the NAC has not been adequately studied or demonstrated to date, and the time/effort-consuming aspects and limited data availability, particularly regarding its usefulness in cases of *Cupressus* allergy, limit its use. For instance, in a recent study from Spain where a total of 71 NACs were performed, the NAC was found to be highly sensitive (100%) but not very specific (15%), except for the 1 IR (69%) concentration [30]. In our study, we used higher allergen extract concentrations in the NAC and patients with positive results showed symptoms at the highest two allergen extract concentrations. This may be a sign of the need to use higher concentrations when performing the NAC and the negative outcomes of the NAC in our study could be a type 2 error. The fact that Cup a1 sIgE values tended to be higher in the NAC+ subgroup also supports this view. Alternatively, it was argued that in a subgroup of children, *Cupressus s*ensitivity might have been an early stage of allergy development and that clinical reactivity could occur over time, which may be more informative if such patients are followed [31].

AR is characterized by paroxysms of sneezing, runny nose, and nasal congestion, often accompanied by itchy eyes, nose, and palate [32–35]. In our study, the majority of patients with *Cupressus* sensitization had other sensitivities, particularly to grass pollen. In clinical practice, it can be difficult to determine which pollen causes symptoms, especially when pollen seasons overlap. According to the available literature from Türkiye, the peak pollen season for *Cupressus* is early spring, while for grass pollen it is late spring and early summer [4]. Therefore, we used an empirical definition for the reporting of *Cupressus* allergy symptoms of AR between February and March, while it was between May and June for grass pollen allergy. When we examined the groups according to NAC outcomes, we found that watery eyes, mouth breathing, sneezing, redness of the eyes, and nasal congestion were more common in the NAC+ subgroup (p < 0.05), which suggests that these symptoms are more suggestive for *Cupressus* allergy diagnosis in the process of asking patients about their symptoms.

The main limitation of this study is that neither Cup a 1 sIgE nor the NAC was evaluated for all patients. Although this is a common limitation of retrospective reviews, our data are sufficient to overturn the assumption that there are few patients with *Cupressus* sensitization in Türkiye and only a minority have a true allergy. Many NACs and component measurements have been applied in clinical practice, but these were necessary to overturn this aforementioned viewpoint and persuade consultants. A final limitation is that this allergy was evaluated in a single center of the country, but it would not be unfair to generalize this finding of an increase on a nationwide level considering that *C. arizonica* has been widely used in plantations and landscaping throughout the country in the last 10 years. The strengths of the study include the fact that it is the first to document an increase in sensitivity and allergy to *Cupressus* in the East Mediterranean region, improve NAC practices with *Cupressus*, and confirm some existing information, such as the reliability of SPTs with *C. arizonica* extract and the increased incidence of *Cupressus* sensitivity/allergies.

In conclusion, the East Mediterranean region is experiencing an increase in *C. arizonica* pollen sensitivity and allergy, which is a continuation of the increase in the European parts of the West and Central Mediterranean regions. Although one of the reasons for this increase is the change in planting and gardening habits, the impact of climate change is undeniable. The *C. arizonica* antigen should be included in aeroallergen skin test panels in the East Mediterranean region.

## Figures and Tables

**Figure:**
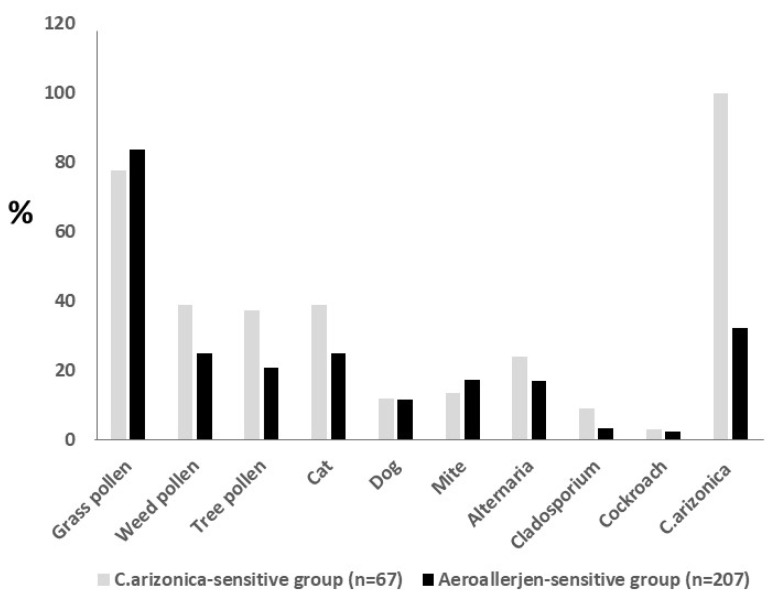
Distribution of aeroallergen sensitivities in the aeroallergen-sensitive and *C. arizonica*-sensitive group.

**Table 1:** Characteristics of the patient group for which component sIgE (Cup a 1) was measured.

	Patients with sIgE measurement (n = 26)
Age, years¶	10.2 (8–14.6)
Sex: male, n (%)	21 (80.8)
Aeroallergen sensitization	
Any house dust mite, n (%)	2 (7.7)
Cat dander, n (%)	11 (42.3)
Dog dander, n (%)	3 (11.5)
Weed pollen mix, n (%)	11 (42.3)
*Alternaria*, n (%)	7 (26.9)
Tree pollen mix, n (%)	11 (42.3)
Grass pollen, n (%)	20 (76.9)
Cockroach, n (%)	1 (3.8)
*Cladosporium*, n (%)	3 (11.5)
Current atopic dermatitis, n (%)	5 (19.2)
Current FA, n (%)	3 (11.5)
Current asthma, n (%)	8 (30.8)
Familial atopy history, n (%)	15 (57.7)
Singleplex arrays, n (%)	10 (38.5)
Multiplex arrays, n (%)	16 (61.5)
NAC performed, n (%)	19 (73)
NAC+ subgroup	10 (38.5)
NAC– subgroup	9 (61.5)
*C. arizonica* wheal edema (mm)¶	6 (4.5–7)
Cup a 1 sIgE (kU/L)¶ (n = 10)	16.5 (2.8–96.9)
ALEX^2^ *Cupressus arizonica* (nCup a 1) sIgE (kU_A_/L)¶ (n = 16)	10.7 (2.6–17.3)
ALEX^2^ *Cryptomeria japonica* (rCry j 1) sIgE (kU_A_/L)¶ (n = 16)	2.9 (0.7–6.4)
Alex *Cupressus sempervirens* (Cup s) sIgE (kU_A_/L)¶ (n = 16)	0.5 (0.15–1.16)

¶ Median, IQR (interquartile range);

NAC: nasal allergen challenge; FA: food allergy; sIgE: specific IgE.

**Table 2:** Characteristics of the patients who underwent nasal allergen challenges.

	NAC group (n = 44)	NAC+ subgroup (n = 13)	NAC– subgroup (n = 31)	p
Age, years¶	12.3 (9.3–14.7)	11.7 (8.9–14)	12.7 (9.4–14.9)	ns
Sex: male, n (%)	32 (72.7)	10 (76.9)	22 (71)	ns
Aeroallergen sensitization				
Any house dust mite, n (%)	6 (13.6)	2 (15.4)	4 (12.9)	ns
Cat dander, n (%)	20 (45.5)	8 (61.5)	12 (38.7)	ns
Dog dander, n (%)	7 (15.9)	2 (15.4)	5 (16.1)	ns
Weed pollen mix, n (%)	19 (43.2)	3 (23.1)	16 (51.6)	ns
*Alternaria*, n (%)	13 (29.5)	2 (15.4)	11 (35.5)	ns
Tree pollen mix, n (%)	15 (34.1)	2 (15.4)	13 (41.9)	ns
Grass pollen, n (%)	33 (75)	10 (76.9)	23 (74.2)	ns
Cockroach, n (%)	2 (4.5)	0	2 (6.5)	ns
*Cladosporium*, n (%)	5 (11.4)	2 (15.4)	3 (9.7)	ns
Current atopic dermatitis, n (%)	10 (22.7)	5 (38.5)	5 (16.1)	ns
Current FA, n (%)	5 (11.4)	1 (7.7)	4 (13)	ns
Current asthma, n (%)	16 (36.4)	4 (30.8)	12 (38.7)	ns
Oral food allergy, n (%)	2 (4.5)	0	2 (6.5)	ns
Allergic rhinitis symptoms, n (%)				
Rhinorrhea	25 (56.8)	10 (76.9)	15 (48.4)	ns
Sneezing	35 (79.5)	13 (100)	22 (71)	0.03
Itchy nose	32 (72.7)	9 (69.2)	23 (74.2)	ns
Nasal congestion	37 (84.1)	12 (92.3)	25 (80.6)	ns
Postnasal drip	11 (25)	5 (38.5)	6 (19.4)	ns
Snoring ± apnea	6 (13.6)	2 (15.4)	4 (12.9)	ns
Watery eyes	26 (59.1)	11 (84.6)	15 (48.4)	0.026
Redness in eyes	26 (59.1)	11 (84.6)	15 (48.4)	0.026
Itchy eyes	27 (61.4)	11 (84.6)	16 (51.6)	0.040
Mouth breathing	9 (20.5)	5 (38.5)	4 (12.9)	0.050
Familial atopy history, n (%)	21 (47.7)	6 (46.2)	15 (48.4)	ns

¶ Median, IQR (interquartile range);

ns: nonsignificant; NAC: nasal allergen challenge; FA: food allergy.

**Table 3:** Laboratory characteristics of the nasal allergen challenge group.

	NAC group (n = 44)	NAC+ subgroup (n = 13)	NAC– subgroup (n = 31)	p
*C. arizonica* wheal edema (mm) ¶	5 (4–6)	6 (5–7.5)	4.5 (4–6)	0.004
WBC, μL/mL ¶	6500 (5950–8110)	6650 (5500–7227.5)	6500 (5950–8300)	ns
Eosinophil (n) ¶	400 (200–525)	435 (200–575)	400 (250–525)	ns
Eosinophil (%)¶	5.6 (3.9–8.1)	6.4 (4.8–9.1)	5.2 (3.4–8.1)	ns
Total IgE (kU/L) ¶	553 (242–1158)	561.5 (245.5–1073)	491 (131–1158)	ns
FEV_1_%¶ ⱡ	94 (87.5–107)	93.5 (89.3–103)	94 (86–109)	ns
FVC%¶ ⱡ	90 (85.5–104)	92 (83.5–102)	90 (87.3–100)	ns
FEF 25%–75%¶ ⱡ	107 (96–123)	103 (96–131)	109 (96.3–120.3)	ns
Cup a 1 sIgE (kU/L) ¶ (n = 10)	16.5 (2.8–96.9)	38.1 (7–122)	3.4 (2.4–82)	ns
ALEX^2^ *Cupressus arizonica* (nCup a 1) sIgE (kU_A_/L) ¶ (n = 9)	3.2 (2–17)	17.1 (5.5–42)	2.4 (1.7–7.4)	ns
ALEX^2^ *Cryptomeria japonica* (rCry j 1) sIgE (kU_A_/L) ¶ (n = 9)	3.1 (0.3–5.1)	3.9 (1.1–26.8)	1.04 (0.2–4.5)	ns
Alex *Cupressus sempervirens* (Cup s) sIgE (kU_A_/L) ¶ (n = 9)	0.56 (0.3–1)	0.96 (0.5–2)	0.5 (0.1–0.6)	ns

¶ Median, IQR (interquartile range); ns: nonsignificant;

ⱡ lung function tests at last visit;

NAC: nasal allergen challenge; WBC: white blood cell count; sIgE: specific IgE; ALEX^2^ sIgE was evaluated for 9 patients; Cupa 1 sIgE was evaluated for 10 patients.
